# Neuroplastic Effects of Transcranial Direct Current Stimulation on Painful Symptoms Reduction in Chronic Hepatitis C: A Phase II Randomized, Double Blind, Sham Controlled Trial

**DOI:** 10.3389/fnins.2015.00498

**Published:** 2016-01-11

**Authors:** Aline P. Brietzke, Joanna R. Rozisky, Jairo A. Dussan-Sarria, Alicia Deitos, Gabriela Laste, Priscila F. T. Hoppe, Suzana Muller, Iraci L. S. Torres, Mário R. Alvares-da-Silva, Rivadavio F. B. de Amorim, Felipe Fregni, Wolnei Caumo

**Affiliations:** ^1^Laboratory of Pain and Neuromodulation, Department of Clinical Research Center, Hospital de Clínicas de Porto Alegre, Universidade Federal do Rio Grande do SulPorto Alegre, Brazil; ^2^Department of Internal Medicine (Gastroenterology/Hepatology), Hospital de Clínicas de Porto Alegre, Universidade Federal do Rio Grande do SulPorto Alegre, Brazil; ^3^Laboratory of Neuromodulation and Center for Clinical Research Learning, Physics and Rehabilitation Department, Spaulding Rehabilitation HospitalBoston, MA, USA

**Keywords:** Hepatitis C, chronic pain, Hepatitis C virus, PEG-IFN, transcranial direct current stimulation

## Abstract

**Introduction:** Pegylated Interferon Alpha (Peg-IFN) in combination with other drugs is the standard treatment for chronic hepatitis C infection (HCV) and is related to severe painful symptoms. The aim of this study was access the efficacy of transcranial direct current stimulation (tDCS) in controlling the painful symptoms related to Peg-IFN side effects.

**Materials and Methods:** In this phase II double-blind trial, twenty eight (*n* = 28) HCV subjects were randomized to receive either 5 consecutive days of active tDCS (*n* = 14) or sham (*n* = 14) during 5 consecutive days with anodal stimulation over the primary motor cortex region using 2 mA for 20 min. The primary outcomes were visual analogue scale (VAS) pain and brain-derived neurotrophic factor (BDNF) serum levels. Secondary outcomes were the pressure-pain threshold (PPT), the Brazilian Profile of Chronic Pain: Screen (B-PCP:S), and drug analgesics use.

**Results:** tDCS reduced the VAS scores (*P* < 0.003), with a mean pain drop of 56% (*p* < 0.001). Furthermore, tDCS was able to enhance BDNF levels (*p* < 0.01). The mean increase was 37.48% in the active group. Finally, tDCS raised PPT (*p* < 0.001) and reduced the B-PCP:S scores and analgesic use (*p* < 0.05).

**Conclusions:** Five sessions of tDCS were effective in reducing the painful symptoms in HCV patients undergoing Peg-IFN treatment. These findings support the efficacy of tDCS as a promising therapeutic tool to improve the tolerance of the side effects related to the use of Peg-IFN. Future larger studies (phase III and IV trials) are needed to confirm the clinical use of the therapeutic effects of tDCS in such condition.

**Trial registration:** Brazilian Human Health Regulator for Research with the approval number CAAE 07802012.0.0000.5327.

## Introduction

Hepatitis C virus (HCV) is the major cause of chronic liver disease, with an estimation of 160–170 million infected people around the world. If not treated appropriately, it can leads to severe consequences, such as development of hepatocellular carcinoma, cirrhosis, and liver failure. In spite of the advent of first-wave generation direct-acting antivirals (DAAs), like telaprevir and boceprevir, the associated use Pegylated Interferon (Peg-INF) in combination with ribavirin and/or protease inhibitors represents the current best therapy recommendation for chronic HCV (Kohli et al., 2014; EASL, 2015).

One of the most relevant extrahepatic adverse outcome is the presence of neurovegetative symptoms. Classically, they are represented by fast and progressive pain development [headaches, myalgia, arthralgia, fatigue, and psychomotor slowing] (Loftis and Hauser, 2004; Huckans et al., 2015) which typically continues over the treatment. The control of pain is often neglected in such patients and is strongly related to a decrease in life-quality and treatment interruption (Louie et al., 2012; Zhanga et al., 2015). The main causes of neurovegetative symptoms are still unclear. Nonetheless, it is a decrease of brain-derived neurotrophic factor (BDNF) in HCV patients treated with Peg-INF (Kenis et al., 2011). BDNF is a neurotrophic factor that is ubiquitously distributed in the CNS and has been recognized as an important marker of neuronal plasticity and is associated with chronic pain. It has a critical role in sensitization and its associated neuroplasticity due to changing excitatory/inhibitory balance in the CNS and increases pain neurotransmission through modulation of nociceptive inputs as well (Chassot et al., 2015). Serum BDNF levels have been shown to be inversely associated with pressure-pain threshold (PPT) in fibromyalgia (Zanette et al., 2014). Similarly, BDNF is negatively correlated with pain scores in chronic myofascial pain syndrome (Dall'Agnol et al., 2014).

Transcranial direct current stimulation (tDCS) is a non-invasive brain stimulation that has revealed promising results in the management of several diseases (Antal et al., 2008; Hansen et al., 2010; Lindenberg et al., 2010; Borckardt et al., 2011; Dasilva et al., 2012; Knotkova et al., 2012; Kumru et al., 2013). It modulates cortical excitability by a low electrical current applied to the brain through electrodes placed on the scalp (Nitsche and Paulus, 2000; Fregni et al., 2005; Dieckhöfer et al., 2006). tDCS has several advantages such as safety, low-cost, and few adverse effects. Moreover, the unique role in controlling and relieving pain has an important clinical role since many RCTs have shown its efficacy in addressing this issue (Nitsche et al., 2007; Wagner et al., 2007).

The mechanisms underlying the side effects induced by Peg-INF are related to neuroinflammatory and neuroplasticity processes. New therapies that can counteract on such mechanisms to guarantee the continuity and optimizing Peg-INF treatment are of utmost importance for a better management of HCV patients (Loftis and Hauser, 2004; Kohli et al., 2014; EASL, 2015; Huckans et al., 2015). Therefore, in order to provide new insights into the neurobiology of the painful symptoms related to Peg-INF in chronic HCV, we performed a phase II randomized clinical trial, double blind, to access whether tDCS could control such symptoms. Moreover, we also hypothesized that tDCS can modulate important neuroplasticity markers in HCV patients, such as BDNF.

## Materials and methods

**Figure 1:**
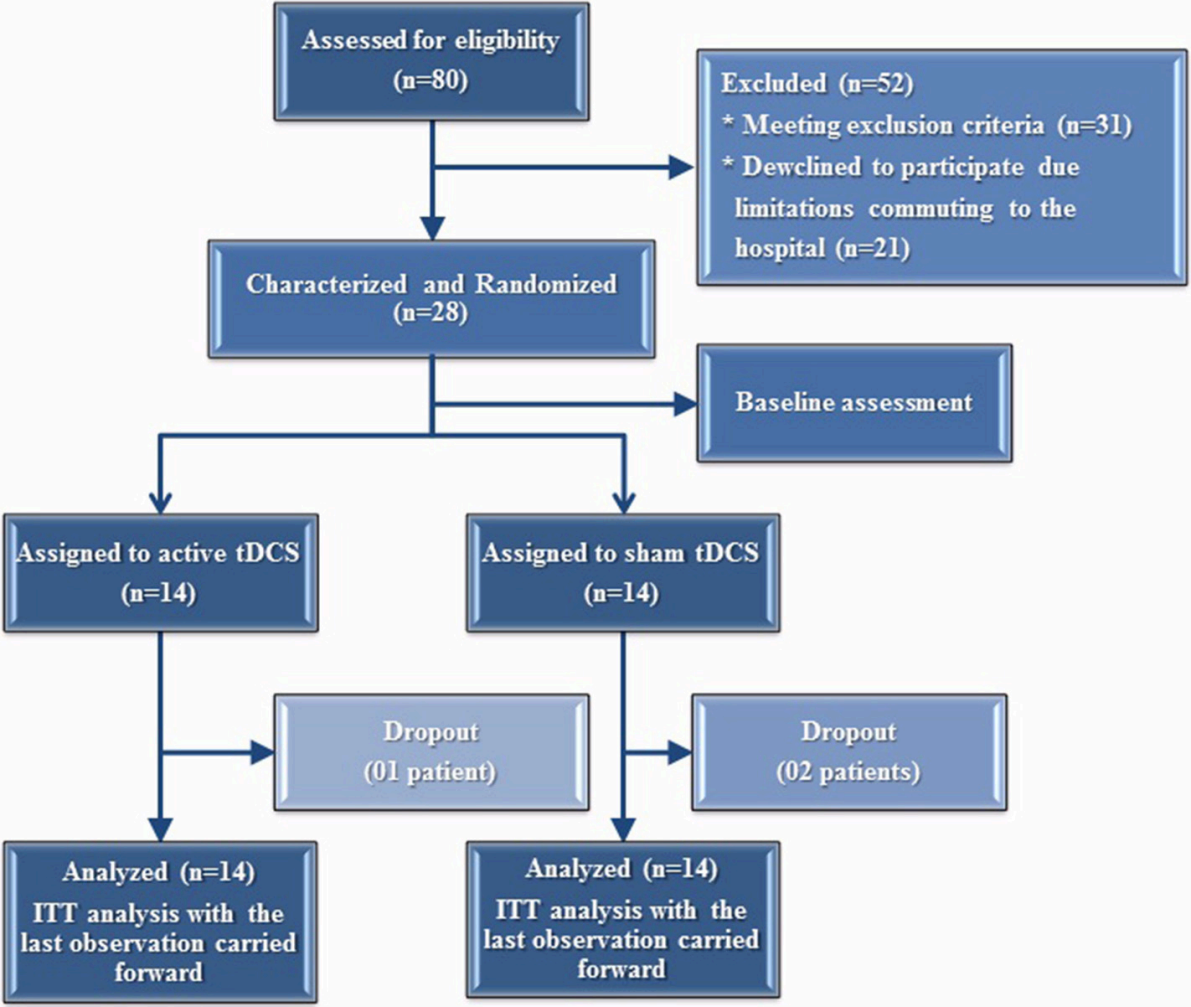
**Randomization and follow-up of the study subjects**.

The current study was designed according to the CONSORT guidelines for transparent reporting of trials. The methodological background chart of the study is shown in Figure 1.

### Study design and eligibility

This phase II randomized, double-blind, sham-controlled, two-arm parallel design, was conducted at one single center study in Hospital de Clínicas de Porto Alegre (Rio Grande do Sul, Brazil). The study was reviewed and approved by the IRB (IRB from the Hospital de Clínicas de Porto Alegre—HCPA/UFRGS/Approval number: 154.287) and conducted in accordance with the Declaration of Helsinki Principles. Written informed consent was obtained from all participants. The trial was registered at the Brazilian Human Health Regulator for Research with the approval number CAAE 07802012.0.0000.5327 (Platform Brazil, aplicacao.saude.gov.br/plataformabrasil/login.jsf).

The studied enrolled 80 subjects in according to the following parameters: (i) Diagnosis of chronic C hepatitis (according to the detection of anti-HCV antibodies and HCV RNA in the presence of histological or biological signs of chronic hepatitis). (ii) Current treatment with Peg-INF in combination with ribavirin and/or protease inhibitors.

### Inclusion and exclusion criteria

In order to be a part of the study, the subjects had to fulfill all of the following inclusion criteria: (i) Age range between 18 and 75 years. (ii) Pain VAS scores ≥ 4. (iii) Presence of depression (assessed by Beck Depression Inventory) for at least 3 months prior of enrollment. (iv) Statement of daily activities limitations (absence from work, decrease of social skills, loss of emotional involvement in routine activities, lack of personal goals, and restriction in cognitive abilities, such as loss of concentrating and memory) related to HCV infection. Participants were excluded if they met the following parameters: (i) History of liver transplant. (ii) Presence of any metal object or implant in brain, skull, scalp, or neck. (iii) Implantable devices, including cardiac pacemakers and defibrillators. (iv) Pregnancy. (v) Morbidly obesity (BMI above 40). (vi) HIV infection. (vii) History of alcohol or substance abuse in the past 6 months. After applying the inclusion and exclusion criteria, 31 patients were excluded. Moreover, 21 patients declined participating due to difficulty in accessing the hospital according to the required study protocol. A final sample size of 28 subjects was randomized.

### Randomization, allocation concealment, implementation, and masking

Randomized numbers in a 1:1 ratio were generated using appropriate software (*www.randomization.com*) to assign each participant to either active or sham-placebo group. The patients were randomized in blocks of four without stratification. Envelopes were prepared for randomization process and sealed. After subject's agreement to participate in the trial, one investigator who was not involved with either stimulation or assessments opened the envelope. The allocation concealment was reached since no investigator (stimulators nor accessors) was aware of treatment allocations and had no control over the order of patients randomized. During the entire protocol timeline, two investigators were responsible for the blinding and randomization procedures.

### Intervention

tDCS was delivered using the anode electrode positioned over the left primary motor cortex (M1) and the cathode electrode at supra orbital right region. The electrodes were placed into a 25–35 cm^2^ square sponge immersed in saline solution for better current conductivity. Current density used was 2 mA and electrodes attached to the scalp were sustained by rubber band. The stimulation period was based on previous studies and the protocol used was 20 min for 5 consecutive days (Boggio et al., 2009; Valle et al., 2009; Knotkova et al., 2012).

### Supplementary analgesic use

Patients were allowed to use supplementary analgesic drugs (acetaminophen, dipyrone) to relieve their pain if necessary. They we asked to record their analgesic intake during the treatment period in pain diaries. The total analgesic dose took during the treatment period was considered for the analysis.

### Instruments and assessments

The psychological tests used in this study had been validated for the Brazilian population (Babor et al., 1989; Kaipper et al., 2010; Sehn et al., 2012; Warmenhoven et al., 2012; Caumo et al., 2013). Two independent blinded examiners were trained to apply the pain scales and to conduct the psychological tests. The baseline depressive symptoms of the patients were assessed using the Beck Depression Inventory (Warmenhoven et al., 2012). The screening test for alcohol misuse is the Alcohol Use Disorders Identification Test (AUDIT) was used to screen for all types of alcohol misuse, i.e., hazardous drinking, harmful drinking, and dependence (Babor et al., 1989).

### Outcomes and assessments

The primary outcomes were VAS pain scores and BDNF serum levels. Secondary outcomes were the PPT and the change of the pain assessed by Brazilian Profile of Chronic Pain: Screen (B-PCP:S) which represents the validated Portuguese version Profile of Chronic Pain: Screen (PCP:S).

### Assessment of outcomes

(i) The intensity of pain was measured by the visual analog scale (VAS). The VAS scores ranged from no pain (zero) to the worst possible pain (Dall'Agnol et al., 2014). Patients were asked to rate their level of pain during related to the last 24 h before the treatment every single day. As soon as the stimulation finished, they were asked to rate again the pain level using VAS scale. (ii) The B-PCP:S in order to identify the individual's multidimensional pain experience. The B-PCP:S addressees three dimensions related to pain (severity, interference, and emotional burden) and it was applied at baseline and at the end of treatment. (iii) PPT. The PPT was examined (baseline and after final session) using an algometer device (*JTECH Medical Industries, Salt Lake City, UT*). The algometer's 1 cm^2^ hard-rubber probe was pressed against the right antecubital fossa with constant increasing pressure. The procedure stopped as soon as the patient indicated uncomfortable pain pressure. (iv) Serum levels of BDNF. Blood samples were collected at baseline and by the end of treatment. Serum BDNF was determined by the Enzyme-Linked Immunoabsorbent Assay (ELISA) using a ChemiKine BDNF Sandwich ELISA Kit, CYT306 (Chemicon/Millipore, Billerica, MA, USA). The lower detection limit of the kit is 7.8 pg/mL for BDNF.

### Sample size method and statistical analysis

An *a priori* estimation indicated a sample size of 24 patients divided into two groups (*n* = 12) in order to detect a 1.5 cm reduction (average standard deviation 0.8 cm) in pain VAS level intensity with a power and α levels of 0.8 and 0.025, respectively. Such a reduction would be clinically relevant and comparable to other pharmacological interventions (Vidor et al., 2012; Caumo et al., 2013; Schwertner et al., 2013). To account for multiple outcomes and considering an attrition rate around 30%, the sample size was increased to 14 patients per group.

The continuous and categorical variables were summarized using conventional descriptive statistics. The *t*-test, chi-square or Fisher's exact tests were used to compare the continuous and the categorical variables, respectively. For non-parametric distributions, group comparisons were performed using the Wilcoxon-Mann-Whitney test. Daily values recorded in the pain scales were averaged to generate one value for each intervention week. The normality assumption for the VAS and the B-PCP:S was tested using the skewness and kurtosis tests. To analyze the effect of the intervention on the VAS, we conducted a group analysis by running a mixed ANOVA model. If appropriate, *post-hoc* analyses considering Bonferroni's adjustment for multiple comparisons were performed. We calculated the adjusted mean differences to access efficacy. The confidence intervals (95% CI) and associated *P*-values were calculated. The standardized mean difference (SMD) was computed in terms of the ratio between the mean change and the placebo-sham standard deviation. The SMD (also known as the effect size) was interpreted as follows: small (≤ 0.40); moderate (0.41–0.79); and high (≥0.80) (Middel and van Sonderen, 2002). An intention-to-treat (ITT) analysis was performed, with the last observation carried forward. The serum BDNF was log transformed and used as dependent variable in a linear regression model including the experimental group (placebo-sham and tDCS) and the cumulative pain score on the VAS as independent variables. The data were analyzed using SPSS, version 18.0 (SPSS, Chicago, IL).

## Results

### Demographic and clinical characteristics of the subjects

A total of 80 subjects were enrolled in this study. After applying the inclusion and exclusion criteria, 31 patients were excluded. In addition, 21 patients declined to participate in the study due to difficulty in accessing the hospital according to the study protocol (5 consecutive days in a week). A final sample size of 28 subjects was randomized. The clinical and demographic characteristics of the patients are presented in Table 1. Fourteen patients were allocated to either active tDCS or placebo-sham. Twenty five patients completed the study; one dropout in the tDCS group two dropouts in the sham group. Baseline features were balanced between the studied groups (*p* > 0.05).

**Table 1:** **Baseline demographic and clinical characteristics of the patients at active or sham group**.

	**Placebo-sham (*n* = 14)**	**tDCS (*n* = 14)**	***p*-value**
Education (years)	9.14 ± 3.14	9.43 ± 3.55	0.86
Age (years)	56.57 ± 8.52	53.86 ± 5.76	0.33
Gender M/F	12/2	9/5	0.2
Beck depression inventory	22.14 ± 7.81	24.57 ± 9.91	0.47
Profile of chronic pain: screen for a brazilian population (B-PCP:S)	70.58 ± 13.27	67.93 ± 12.65	0.49
Brain derived neurotrophic factor (BDNF) levels	13 (74)	13.83 (6.60)	0.36
	11.72 (7.48–20.35)	12.03 (9.97–20.29)	
Pain on visual analogue scale (VAS)	7.38 (1.19)	6.81 (1.81)	0.33
Pain pressure threshold (PPT)	4.42 (1.44)	2.77 (1.04)	0.18

### Analysis of the primary outcomes: VAS scores and serum BDNF

**Figure 2:**
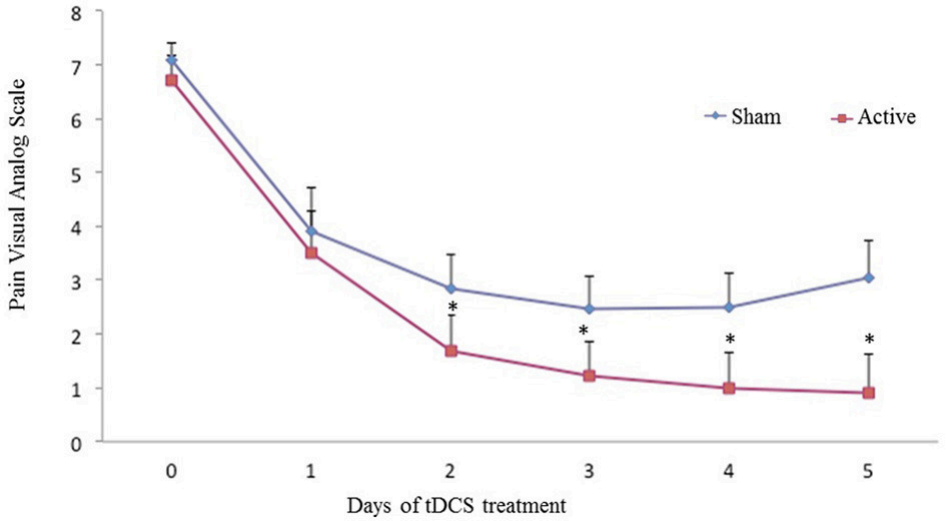
**Pain VAS scores at baseline and during the treatment**. The error bars indicate the standard error of the mean (SEM). Asterisks (^*^) positioned above the symbols indicate significant differences (*p* < 0.01) at those time points using Anova model type III test.

The active-tDCS group had significantly lower pain VAS scores (*P* < 0.003) starting at the second session until the end of the treatment (Figure 2). The interaction between time and treatment group was not significant (*P* = 0.07). There was no interaction between time and the intervention group (*P* = 0.07). The cumulative mean ± SD pain on the VAS was 1.68 ± 2.31 in the group receiving tDCS and 3.0 ± 2.74 in the placebo-sham. The tDCS group had a mean pain reduction of 56% (*P* < 0.001) compared with placebo-sham, representing a moderately sized effect (Cohen's *d* = 0.49).

**Figure 3:**
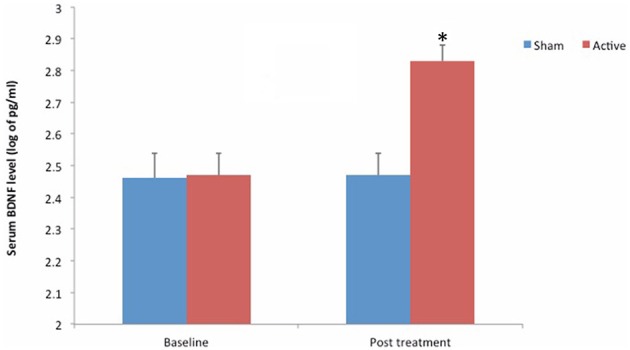
**Mean serum BDNF levels at baseline and after treatment**. Asterisk (^*^) positioned above the symbols indicate significant differences (*p* < 0.01).

Concerning BDNF, the tDCS group had a significantly higher serum levels (*P* < 0.01) (Figure 3). The mean increase from the baseline was 37.48% in the tDCS-active group, whereas the placebo-sham group presented a mean reduction of 1.48%. To address whether the BDNF reduction was secondary to pain improvement or a primary effect of the intervention, we conducted an additional regression model. The adjusted mean difference in the BDNF level between the tDCS and the placebo-sham groups was of 4.64 (95% CI = 2.3–7.07, *P* < 0.001). The interaction between the intervention group and pain on the VAS was significant (*P* < 0.01), indicating that the variability in the serum BDNF was related to the pain and the intervention group. In stratifying by the intervention groups, the effect of pain was significant for both groups (Table 2). The correlation was inversed in the placebo-sham group, which means that the pain increase was correlated with the lower serum BDNF.

**Table 2:** **Multivariate linear regression of the pain reported compared with BDNF, treatment group and daily pain VAS (***n*** = 24)**.

**Parameter**	**B**	**T**	***P*-value**	**95% CI**
**DEPENDENT VARIABLE: SERUM BDNF IN THE END OF TREATMENT**				
Cumulative worst pain score on VAS diary (mean of 5 days)	−1.54	−2.87	0.005	(−2.59 to −0.48)
Placebo-sham ^*^ (Cumulative worst pain score on VAS) vs.	−0.49	−0.32	0.74	(−3.43 to 2.46)
Active-tDCS ^*^ (Cumulative worst pain score on VAS) vs.				
Interaction	1.05	2.98	0.003	(0.35 to 1.74)
Cumulative worst pain score on VAS diary ^*^ (placebo-sham)	−0.49	−2.0	0.02	(−0.98 to −0.04)
Cumulative worst pain score on VAS diary ^*^ (active-tDCS)	0.28	2.25	0.03	(0.03 to 0.52)

*BDNF, brain derived neurotropic factor; VAS, visual analogue scale; CI, confidence interval*.

*Linear regression model—Adjusted R^2^ = 0.10*.

* *Numerical Pain Scale collected before and after tDCS during all days of treatment*.

### Secondary outcomes: PPT, analgesic use, and B-PCP:S score

**Figure 4:**
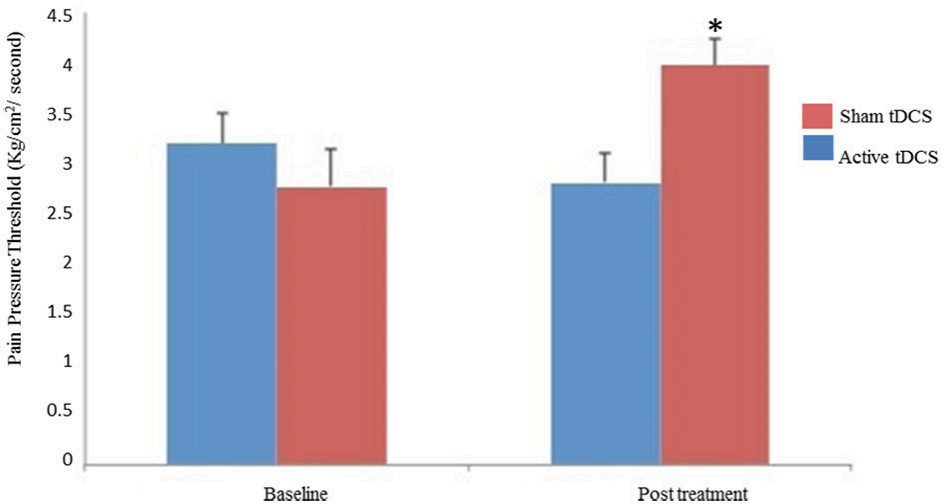
**Mean pain threshold at baseline and at the end of treatment**. The error bars indicate the standard error of the mean (SEM). Asterisks (^*^) positioned above the symbols indicate significant differences (*p* < 0.001).

In the active tDCS group, the adjusted mean (±SD) of the PPT at the end of treatment was 3.97 ± 1.34 (43.32% mean increase from baseline) as compared with 2.99 ± 1.11 (2.68% mean reduction from baseline) (*p* = 0.007) (Figure 4). The end of treatment PPT was adjusted for baseline levels (r-squared = 0.46, standard β coefficient for the baseline pain threshold = 0.79, *t* = 4.01), standard β coefficient for the active-tDCS was 1.15 (placebo-sham was reference group), *t* = 2.97, both *P* < 0.001.

The B-PCP:S was lower in the tDCS group with 29.07 ± 9.20, compared to 50.58 ± 14.33 in the placebo-sham group (*P* < 0.001). The mean difference at the end of the intervention was 21.50 [confidence interval (CI) 95% = 12.02–30.99], indicating that the tDCS treatment effect size, on the B-PCP:S, was large (i.e., Cohen's *d* = 1.50).

Analgesic use during the intervention period occurred in 63% of the patients in the placebo-sham group and in 37% of the patients in the active-tDCS group. The relative risk for using analgesics during the 5 days of treatment was 1.43 (95% CI 1.04–1.96); the placebo-sham group was 43% more likely to require additional analgesics. There was a significant reduction in the number of analgesic doses for the patients receiving tDCS compared to those receiving the placebo-sham (*p* < 0.03).

## Discussion

This current study showed that tDCS reduced the painful symptoms induced by Peg-IFN in patients with chronic HCV compared to a placebo-sham. The effect was statistically significant and has clinical relevance taking into account our findings. Moreover, tDCS treatment was associated with increased BDNF serum levels and also with an improvement at the PPT test. To our knowledge and based on a careful review of the scientific literature (Pubmed, Web of science, Scopus, and Isi) this is the first evidence showing tDCS positive effects in controlling pain in HCV infected patients undergoing Peg-IFN therapy.

As mentioned before, the mechanisms underlying the etiology of Peg-IFN painful symptoms are not well known. It is believed that a trigger activates the system network that stimulates the central nervous system (CNS) inflammatory pathways (Raison et al., 2009). The tDCS effect on pain might be related to an interruption in the response associated with maladaptive neuroplasticity, which induces neuroimmune reactions that could amplify pain signals in the neural pain matrix (Xanthos and Sandkühler, 2014). In addition, tDCS may inhibit microglial activation observed in inflammatory diseases such as viral infections and the neuroinflammatory reactions induced by Peg-IFN (Par et al., 2013).

Our findings suggest that tDCS induces neuroplastic changes in pain pathways, including modulation in neurotransmitters that regulate pain (i.e., it increases the serum BDNF levels). Especially, our results were related to the pain symptoms status of the subjects indicating the clinical relevance of tDCS therapy in such scenario. Some studies indicate that the Peg-INF is responsible for activating CNS inflammatory pathways by stimulating and releasing proinflammatory in both periphery and CNS (Quan and Banks, 2007; Raison et al., 2009). The relationship between the serum BDNF and pain in the current study provides evidence of the role of Peg-IFN in the sensitization of the CNS areas associated with nociceptive sensory processing. Considering the increased level of BDNF expression in response to higher levels of neuronal activity, it can be inferred a possible relation with cortical activity that strengthens inhibitory synapses for pain (Genoud et al., 2004). Hence, BDNF could act as a molecular marker of global neuronal activity (Genoud et al., 2004). These findings are in agreement with the tDCS clinical effects demonstrated in the treatment of other types of chronic pain due to different etiologies (Fregni et al., 2006a; Antal et al., 2008; Fenton et al., 2009).

In addition, the decrease of the symptoms of central sensitization induced by Peg-IFN, and its association with the increase BDNF after 5 days of stimulation can lead to some other deductions. For instance, the central sensitization was properly modulated and this was not related to the use of common analgesics. Finally, the tDCS could had reached effects not only in CNS but also in periphery pain pathways, taking into account that the sensitization is orchestrated by neuronal, endocrinal, and immune mechanisms capable of amplifying sensory pain signals to the neural pain matrix (Garcia-Larrea and Peyron, 2007).

In the present study, the tDCS analgesia was consistently demonstrated by different outcomes: reduction on VAS pain scores, decrease on analgesic drugs use, and PPT increase. It must be highlighted that synaptic plasticity processes have a direct connection with the primary somatosensory cortex network, like the neuronal inactivation induced by stimulation of the M1 area. The tDCS can also modulates other structures related to pain facilitation such as the thalamus and the brainstem nuclei, which down regulate processing from the sensitized neurons (Polanía et al., 2011). The tDCS device was operated with a ramp up of 15 s while starting the stimulation (either sham or active) and a ramp down of 15 s during the end of the procedure. A decrease in mean pain scores (3.0 ± 2.74) seen in the placebo-sham group was observed, in spite of a significantly higher reduction in pain scores reported by the tDCS group. Taking into account such findings, it can be mentioned that the placebo effect was detected in our study and that even with sham stimulation the subjects experienced an improvement in pain symptoms (Wechsler et al., 2011). While there is some evidence that placebo interventions can alter levels of hormones (Kokkotou et al., 2010), endocannabinoids or endogenous opioids (Benedetti et al., 2005), other reasons could be related to the Hawthorne effect, regression to the mean and etc. (Barnett et al., 2005).

The strengths of the study include the study design and the major concern of running a RCT that could cover as most as possible the clinical research requirements for high quality trials. The comparison between active tDCS and a placebo-sham intervention in a randomized, two-arm parallel, double blinded, properly masking, and allocation concealment must be stressed. Furthermore, multiple efficacy outcomes gives support to our findings toward the effect of the tDCS and enabled a better knowledge underlying its mechanisms of action. Nonetheless, this study has some limitations. Although the BDNF is secreted by neurons and neuroglia in the CNS (Savli et al., 2004), it actively crosses the blood-brain barrier, contributing to 70–80% of its serum concentration (Schinder and Poo, 2000). We did not assess the possible influence of BDNF polymorphisms on this sample, and they might have an influence in neuronal plasticity. The short follow-up period is another limitation of the current study that should be mentioned. Our study design and framework (2 mA, 20 min, 5 consecutive days, M1 target) were based on parameters reported by some previous studies (Fregni et al., 2006a; Boggio et al., 2009; Valle et al., 2009; Knotkova et al., 2012; Sakrajai et al., 2014; Souto et al., 2014; Fagerlund et al., 2015). One interesting study evaluated the tDCS effect for reducing pain due to spinal cord injury and the cumulative analgesic effects lasted up to 2 weeks after stimulation (Fregni et al., 2006a). The same protocol was adopted in two studies with fibromyalgia and a significant improvement in pain was detected following active tDCS (Fregni et al., 2006b; Fagerlund et al., 2015). In one of these studies, the pain relief effects lasted up to 3 weeks (Fregni et al., 2006b). A recent study using tDCS to reduce myofascial pain (Sakrajai et al., 2014) was able to reduce the pain symptoms and the effects persisted 1 week after the final tDCS session. Considering these data on the lasting effects of tDCS regarding pain relief (2 weeks in average), it can be hypothesized that patients with HCV hepatitis receiving Peg-IFN should have 5 consecutive sessions every 15 days. Further studies with a well-designed follow-up period are needed to evaluate the long lasting effects of tDCS for pain improvement in HCV subjects undergoing Peg-INF treatment. Such data would bring valuable knowledge strengthening the use of tDCS in clinical practice.

To sum up, we have demonstrated the clinical efficacy of tDCS in relieving pain, the increase on PPT increase and BDNF serum levels and the reduction of analgesic use in chronic HCV infected patients receiving Peg-INF. From a clinical standpoint, these findings support the use of tDCS as a promising therapeutic tool to improve the tolerance of the side effects related to the use of Peg-IFN and further studies (phase III and IV trials) are required in order to allow the clinical use of tDCS in as a valid treatment in these patients.

## Author contributions

AB, JR had substantial contributions to the conception or design of the work. AD, GL, JD, PH drafting the work or revising it critically for important intellectual content. WC, Rd had final approval of the version to be published. SM, Id, MA, FF agreement to be accountable for all aspects of the work in ensuring that questions related to the accuracy or integrity of any part of the work are appropriately investigated and resolved.

### Conflict of interest statement

The authors declare that the research was conducted in the absence of any commercial or financial relationships that could be construed as a potential conflict of interest.
